# Ethyl 1-[2-(morpholin-4-yl)eth­yl]-2-[4-(morpholin-4-yl)phen­yl]-1*H*-1,3-benzimidazole-5-carboxyl­ate

**DOI:** 10.1107/S1600536811023294

**Published:** 2011-06-22

**Authors:** Yeong Keng Yoon, Mohamed Ashraf Ali, Rusli Ismail, Mohd Mustaqim Rosli, Hoong-Kun Fun

**Affiliations:** a Institute for Research in Molecular Medicine, Universiti Sains Malaysia, 11800 USM, Penang, Malaysia; b X-ray Crystallography Unit, School of Physics, Universiti Sains Malaysia, 11800 USM, Penang, Malaysia

## Abstract

The asymmetric unit of the title compound, C_26_H_32_N_4_O_4_, consists of two independent mol­ecules. In both mol­ecules, the eth­oxy groups are each disordered over two sets of sites with occupancies of 0.695 (4):0.305 (4) and 0.877 (2):0.123 (2). The dihedral angles between the benzimidazole ring system and the adjacent benzene ring in the two mol­ecules are 41.41 (5) and 31.46 (5)°. In the crystal, mol­ecules are linked by C—H⋯O and C—H⋯N inter­actions.

## Related literature

For biological activity of benzimidazole derivatives, see: Vijaya *et al.* (2009 ▶); Haugwitz (1982 ▶); Hisano (1982 ▶). For the stability of the temperature controller used in the data collection, see: Cosier & Glazer (1986 ▶).
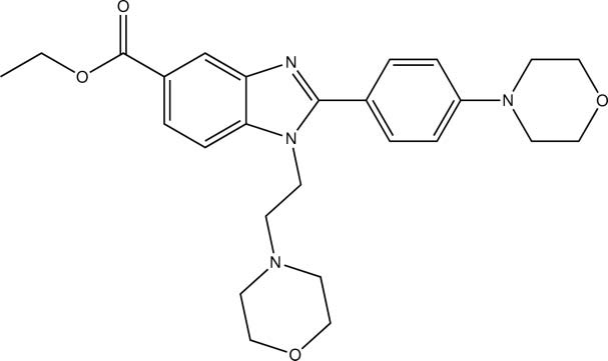


## Experimental

### 

#### Crystal data

C_26_H_32_N_4_O_4_
*M* *_r_* = 464.56
Monoclinic, 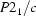

*a* = 10.3595 (3) Å
*b* = 20.6870 (6) Å
*c* = 23.7904 (7) Å
β = 112.839 (2)°
*V* = 4698.7 (2) Å^3^
*Z* = 8
Mo *K*α radiation
μ = 0.09 mm^−1^
*T* = 100 K
0.50 × 0.27 × 0.14 mm

#### Data collection

Bruker SMART APEXII CCD area-detector diffractometer
Absorption correction: multi-scan (*SADABS*; Bruker, 2009 ▶) *T*_min_ = 0.957, *T*_max_ = 0.987
100050 measured reflections
20532 independent reflections
13488 reflections with *I* > 2σ(*I*)
*R*_int_ = 0.042

#### Refinement

*R*[*F* ^2^ > 2σ(*F* ^2^)] = 0.054
*wR*(*F* ^2^) = 0.154
*S* = 1.02
20532 reflections
667 parameters
78 restraints
H-atom parameters constrained
Δρ_max_ = 0.45 e Å^−3^
Δρ_min_ = −0.34 e Å^−3^

### 

Data collection: *APEX2* (Bruker, 2009 ▶); cell refinement: *SAINT* (Bruker, 2009 ▶); data reduction: *SAINT*; program(s) used to solve structure: *SHELXTL* (Sheldrick, 2008 ▶); program(s) used to refine structure: *SHELXTL*; molecular graphics: *SHELXTL*; software used to prepare material for publication: *SHELXTL* and *PLATON* (Spek, 2009 ▶).

## Supplementary Material

Crystal structure: contains datablock(s) global, I. DOI: 10.1107/S1600536811023294/is2728sup1.cif

Structure factors: contains datablock(s) I. DOI: 10.1107/S1600536811023294/is2728Isup2.hkl

Supplementary material file. DOI: 10.1107/S1600536811023294/is2728Isup3.cml

Additional supplementary materials:  crystallographic information; 3D view; checkCIF report

## Figures and Tables

**Table 1:** Hydrogen-bond geometry (Å, °)

*D*—H⋯*A*	*D*—H	H⋯*A*	*D*⋯*A*	*D*—H⋯*A*
C14*B*—H14*D*⋯O4*A*^i^	0.99	2.39	3.1322 (17)	132
C16*B*—H16*C*⋯O1*B*^ii^	0.99	2.42	3.2944 (15)	147
C17*B*—H17*C*⋯O3*A*^iii^	0.99	2.60	3.4963 (18)	151
C23*B*—H23*D*⋯O2*A*^iv^	0.99	2.53	3.3839 (16)	144
C24*B*—H24*D*⋯N1*B*^v^	0.99	2.61	3.4668 (14)	145
C25*B*—H25*C*⋯N3*A*	0.99	2.61	3.5189 (18)	153

Symmetry codes: (i) 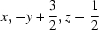; (ii) 
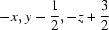
; (iii) 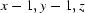; (iv) 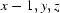; (v) 
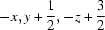
.

**Fig. 1.**
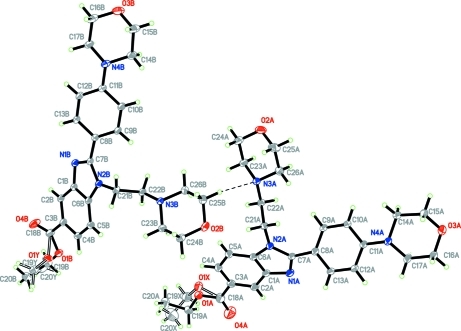
The asymmetric unit of the title compound, showing 50% probability displacement ellipsoids and the atom-numbering scheme. Hydrogen atoms are shown as spheres of arbitrary radius.

**Fig. 2.**
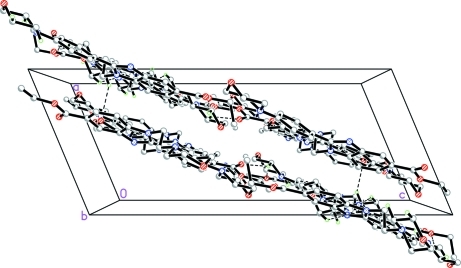
The crystal packing of the title compound, viewed along the b axis. Dashed lines indicate hydrogen bonds. H atoms not involved in the hydrogen bond interactions have been omitted for clarity.

## supplementary crystallographic information

### Comment

The benzimidazole nucleus is an important heterocyclic ring because of its synthetic utility and broad range of pharmacological activities. Benzimidazole derivatives exhibit different pharmacological effects, including antifungal. In recent years, attention has increasingly been given to the synthesis of benzimidazole derivatives as a source of new antimicrobial agents (Vijaya *et al.*, 2009). Recent observations suggest that substituted benzimidazoles and heterocyclic, which are the structural isosters of nucleotides owing to the fused heterocyclic nuclei in their structures that allow them to interact easily with the biopolymers, possess potential activity with lower toxicities in the chemotherapeutic approach in man (Haugwitz, 1982; Hisano, 1982).

In the asymmetric unit (Fig. 1) of the title compound there are two molecules. In both molecules, the ethoxy group were disordered over two position with the refined occupancies of 0.695:0.305 and 0.877:0.123, respectively, for the molecules A and B. The benzimidazole (N1/N2/C1–C7) ring in both molecules is planar with the maximum deviation of 0.0129 (12) from atom C5A and -0.0246 (12) from atom C2B. It makes dihedral angles of 41.41 (5)° and 31.46 (5)° with the adjacent benzene ring, respectively, for molecule A and B. All the four morpholine rings, O2A/N3A/C23A–C26A, O3A/N4A/C14A–C17A, O2B/N3B/C23B–C26B and O3B/N4B/C14B–C17B, adopt chair conformations with [Q = 0.5722 (13)Å, θ = 1.51 (13)° & φ = 127 (5)°], [Q = 0.5411 (12)Å, θ = 8.99 (12)° & φ = 354.4 (9)], [Q = 0.5787 (12)Å, θ = 177.48 (12)° & φ = 58 (3)] and [Q = 0.5389 (14)Å, θ = 170.19 (14)° & φ = 182.8 (10)°], respectively.

In the crystal structure, molecules A and B are linked by C25B—H25C···N3A hydrogen bond. The molecules are further linked by C14B—H14D···O4A^i^, C17B—H17C···O3A^iii^, C23B—H23D···O2A^iv^ and C24B—H24D···N1B^v^ hydrogen bonds (Table 1) to form layers that are connected by a C16B—H16C···O1B^ii^ interaction (Fig. 2).

### Experimental

Ethlyl-3-amino-4-(morpholinoethylamino) benzoate (0.01 mol) and sodium metabisulfite adduct of 4-morpholino benzaldehyde (0.01 mol) were dissolved in DMF. The reaction mixture was refluxed at 130 °C for 4 hrs. After completion, the reaction mixture was diluted in ethyl acetate (20 mL) and washed with water (20 mL). The organic layer was collected, dried over Na_2_SO_4_ and the evaporated in vacuo to yield the product. The product was recrystallized from ethyl acetate.

### Refinement

The ethoxy group was disordered over two position with the refined occupancies of 0.695 (4):0.305 (4) and 0.877 (2):0.123 (2). The same *U*_ij_ parameters were applied to the C19Y/C19B pair. Similarity restraints were used on the disordered part of the molecular structure. All H-atoms were positioned geometrically and refined using a riding model, with C—H = 0.95–0.99 Å, and with *U*_iso_(H) = 1.2*U*_eq_(C) or 1.5*U*_eq_(C_methyl_).

### Crystal data

**Table:**

C_26_H_32_N_4_O_4_	*F*(000) = 1984
*M**_r_* = 464.56	*D*_x_ = 1.313 Mg m^−^^3^
Monoclinic, *P*2_1_/*c*	Mo *K*α radiation, λ = 0.71073 Å
Hall symbol: -P 2ybc	Cell parameters from 9803 reflections
*a* = 10.3595 (3) Å	θ = 3.0–34.4°
*b* = 20.6870 (6) Å	µ = 0.09 mm^−^^1^
*c* = 23.7904 (7) Å	*T* = 100 K
β = 112.839 (2)°	Block, colourless
*V* = 4698.7 (2) Å^3^	0.50 × 0.27 × 0.14 mm
*Z* = 8	

### Data collection

**Table:**

Bruker SMART APEXII CCD area-detector diffractometer	20532 independent reflections
Radiation source: fine-focus sealed tube	13488 reflections with *I* > 2σ(*I*)
graphite	*R*_int_ = 0.042
φ and ω scans	θ_max_ = 34.9°, θ_min_ = 1.9°
Absorption correction: multi-scan (*SADABS*; Bruker, 2009)	*h* = −16→16
*T*_min_ = 0.957, *T*_max_ = 0.987	*k* = −33→33
100050 measured reflections	*l* = −38→38

### Refinement

**Table:**

Refinement on *F*^2^	Primary atom site location: structure-invariant direct methods
Least-squares matrix: full	Secondary atom site location: difference Fourier map
*R*[*F*^2^ > 2σ(*F*^2^)] = 0.054	Hydrogen site location: inferred from neighbouring sites
*wR*(*F*^2^) = 0.154	H-atom parameters constrained
*S* = 1.02	*w* = 1/[σ^2^(*F*_o_^2^) + (0.072*P*)^2^ + 0.8073*P*] where *P* = (*F*_o_^2^ + 2*F*_c_^2^)/3
20532 reflections	(Δ/σ)_max_ = 0.001
667 parameters	Δρ_max_ = 0.45 e Å^−^^3^
78 restraints	Δρ_min_ = −0.34 e Å^−^^3^

### Special details

**Table:**

Experimental. The crystal was placed in the cold stream of an Oxford Cryosystems Cobra open-flow nitrogen cryostat (Cosier & Glazer, 1986) operating at 100.0 (1) K.
Geometry. All esds (except the esd in the dihedral angle between two l.s. planes) are estimated using the full covariance matrix. The cell esds are taken into account individually in the estimation of esds in distances, angles and torsion angles; correlations between esds in cell parameters are only used when they are defined by crystal symmetry. An approximate (isotropic) treatment of cell esds is used for estimating esds involving l.s. planes.
Refinement. Refinement of F^2^ against ALL reflections. The weighted R-factor wR and goodness of fit S are based on F^2^, conventional R-factors R are based on F, with F set to zero for negative F^2^. The threshold expression of F^2^ > 2sigma(F^2^) is used only for calculating R-factors(gt) etc. and is not relevant to the choice of reflections for refinement. R-factors based on F^2^ are statistically about twice as large as those based on F, and R- factors based on ALL data will be even larger.

### Fractional atomic coordinates and isotropic or equivalent isotropic displacement parameters (Å^2^)

**Table:**

	*x*	*y*	*z*	*U*_iso_*/*U*_eq_	Occ. (<1)
O1A	0.2415 (2)	0.93334 (9)	0.95927 (9)	0.0270 (4)	0.695 (4)
C19A	0.2018 (2)	0.94515 (10)	1.01053 (9)	0.0299 (5)	0.695 (4)
H19A	0.1307	0.9799	1.0002	0.036*	0.695 (4)
H19B	0.2847	0.9592	1.0464	0.036*	0.695 (4)
C20A	0.1435 (3)	0.88436 (12)	1.02530 (10)	0.0355 (5)	0.695 (4)
H20A	0.1115	0.8927	1.0583	0.053*	0.695 (4)
H20B	0.2164	0.8510	1.0382	0.053*	0.695 (4)
H20C	0.0644	0.8695	0.9891	0.053*	0.695 (4)
O1X	0.2849 (6)	0.9144 (2)	0.9683 (2)	0.0293 (9)	0.305 (4)
C19X	0.2621 (6)	0.9137 (3)	1.0258 (2)	0.0365 (13)	0.305 (4)
H19C	0.2934	0.8720	1.0470	0.044*	0.305 (4)
H19D	0.3165	0.9488	1.0530	0.044*	0.305 (4)
C20X	0.1115 (6)	0.9230 (4)	1.0108 (3)	0.0565 (19)	0.305 (4)
H20D	0.0932	0.9205	1.0482	0.085*	0.305 (4)
H20E	0.0583	0.8891	0.9824	0.085*	0.305 (4)
H20F	0.0826	0.9654	0.9918	0.085*	0.305 (4)
O4A	0.32655 (15)	1.03045 (5)	0.97339 (5)	0.0584 (4)	
C18A	0.30702 (15)	0.98036 (6)	0.94559 (6)	0.0340 (3)	
O2A	0.65322 (9)	0.72303 (4)	0.62040 (4)	0.03086 (19)	
O3A	0.88432 (10)	1.22442 (4)	0.54127 (4)	0.02935 (19)	
N1A	0.50149 (10)	1.05538 (4)	0.79633 (4)	0.02112 (18)	
N2A	0.44017 (10)	0.96405 (4)	0.74025 (4)	0.01775 (16)	
N3A	0.53247 (10)	0.81363 (4)	0.67653 (4)	0.01855 (17)	
N4A	0.70676 (10)	1.15637 (4)	0.58792 (4)	0.01836 (16)	
C1A	0.43668 (12)	1.01324 (5)	0.82253 (5)	0.01989 (19)	
C2A	0.40671 (13)	1.02074 (5)	0.87442 (5)	0.0235 (2)	
H2AA	0.4318	1.0591	0.8981	0.028*	
C3A	0.33901 (13)	0.97032 (5)	0.89054 (5)	0.0240 (2)	
C4A	0.29955 (12)	0.91355 (5)	0.85502 (5)	0.0234 (2)	
H4AA	0.2534	0.8800	0.8672	0.028*	
C5A	0.32675 (12)	0.90580 (5)	0.80282 (5)	0.0218 (2)	
H5AA	0.2985	0.8681	0.7783	0.026*	
C6A	0.39752 (11)	0.95603 (5)	0.78793 (5)	0.01851 (19)	
C7A	0.50214 (11)	1.02455 (5)	0.74764 (5)	0.01805 (18)	
C8A	0.55510 (11)	1.05466 (5)	0.70493 (5)	0.01805 (18)	
C9A	0.63634 (11)	1.02339 (5)	0.67810 (5)	0.01807 (18)	
H9AA	0.6562	0.9786	0.6856	0.022*	
C10A	0.68876 (11)	1.05649 (5)	0.64056 (5)	0.01800 (18)	
H10A	0.7451	1.0341	0.6236	0.022*	
C11A	0.65970 (11)	1.12262 (4)	0.62729 (5)	0.01684 (18)	
C12A	0.57768 (11)	1.15376 (5)	0.65446 (5)	0.01919 (19)	
H12A	0.5563	1.1984	0.6466	0.023*	
C13A	0.52778 (11)	1.12075 (5)	0.69227 (5)	0.01935 (19)	
H13A	0.4735	1.1433	0.7102	0.023*	
C14A	0.80102 (12)	1.12200 (5)	0.56573 (5)	0.0222 (2)	
H14A	0.7576	1.0805	0.5472	0.027*	
H14B	0.8897	1.1122	0.6005	0.027*	
C15A	0.83236 (15)	1.16150 (5)	0.51881 (6)	0.0281 (2)	
H15A	0.9026	1.1385	0.5076	0.034*	
H15B	0.7458	1.1659	0.4816	0.034*	
C16A	0.78096 (13)	1.25783 (5)	0.55526 (5)	0.0243 (2)	
H16A	0.6941	1.2611	0.5180	0.029*	
H16B	0.8142	1.3022	0.5689	0.029*	
C17A	0.74900 (13)	1.22394 (5)	0.60451 (5)	0.0247 (2)	
H17A	0.8330	1.2249	0.6431	0.030*	
H17B	0.6727	1.2471	0.6113	0.030*	
C21A	0.42635 (11)	0.91451 (5)	0.69454 (5)	0.01818 (19)	
H21A	0.4371	0.9344	0.6588	0.022*	
H21B	0.3321	0.8947	0.6807	0.022*	
C22A	0.53814 (12)	0.86238 (5)	0.72165 (5)	0.01854 (19)	
H22A	0.6319	0.8828	0.7376	0.022*	
H22B	0.5242	0.8413	0.7562	0.022*	
C23A	0.59041 (14)	0.75174 (5)	0.70504 (5)	0.0257 (2)	
H23A	0.5384	0.7363	0.7297	0.031*	
H23B	0.6898	0.7575	0.7326	0.031*	
C24A	0.57951 (14)	0.70230 (5)	0.65659 (6)	0.0309 (3)	
H24A	0.6186	0.6606	0.6763	0.037*	
H24B	0.4797	0.6954	0.6301	0.037*	
C25A	0.59904 (16)	0.78334 (6)	0.59223 (6)	0.0337 (3)	
H25A	0.5001	0.7777	0.5641	0.040*	
H25B	0.6526	0.7977	0.5679	0.040*	
C26A	0.60758 (14)	0.83473 (5)	0.63877 (5)	0.0261 (2)	
H26A	0.7069	0.8431	0.6650	0.031*	
H26B	0.5660	0.8755	0.6176	0.031*	
O1B	−0.19358 (12)	0.57969 (4)	0.96297 (5)	0.0219 (2)	0.877 (2)
C19B	−0.22170 (16)	0.57394 (7)	1.01799 (6)	0.0227 (3)	0.877 (2)
H19E	−0.1861	0.6128	1.0436	0.027*	0.877 (2)
H19F	−0.1715	0.5358	1.0416	0.027*	0.877 (2)
C20B	−0.37591 (16)	0.56685 (7)	1.00312 (7)	0.0288 (3)	0.877 (2)
H20G	−0.3915	0.5614	1.0409	0.043*	0.877 (2)
H20H	−0.4117	0.5289	0.9770	0.043*	0.877 (2)
H20I	−0.4251	0.6056	0.9817	0.043*	0.877 (2)
O1Y	−0.2819 (8)	0.5744 (3)	0.9371 (3)	0.0205 (16)	0.123 (2)
C19Y	−0.3365 (11)	0.5667 (4)	0.9848 (4)	0.0227 (3)	0.123 (2)
H19G	−0.3843	0.5243	0.9799	0.027*	0.123 (2)
H19H	−0.4064	0.6009	0.9806	0.027*	0.123 (2)
C20Y	−0.2242 (17)	0.5704 (6)	1.0454 (6)	0.055 (4)	0.123 (2)
H20J	−0.2642	0.5672	1.0765	0.082*	0.123 (2)
H20K	−0.1749	0.6116	1.0498	0.082*	0.123 (2)
H20L	−0.1584	0.5347	1.0506	0.082*	0.123 (2)
O2B	0.11661 (9)	0.81572 (4)	0.64074 (4)	0.02732 (18)	
O3B	0.39165 (11)	0.26192 (4)	0.53919 (4)	0.0364 (2)	
O4B	−0.19219 (12)	0.47120 (4)	0.95706 (4)	0.0388 (2)	
N1B	0.00759 (10)	0.44467 (4)	0.78758 (4)	0.01896 (17)	
N2B	−0.03377 (9)	0.53852 (4)	0.73501 (4)	0.01702 (16)	
N3B	0.07264 (9)	0.68721 (4)	0.67357 (4)	0.01652 (16)	
N4B	0.22233 (10)	0.33681 (4)	0.58525 (4)	0.02010 (17)	
C1B	−0.05338 (11)	0.48833 (5)	0.81388 (5)	0.01751 (18)	
C2B	−0.09119 (11)	0.48028 (5)	0.86352 (5)	0.01917 (19)	
H2BA	−0.0785	0.4400	0.8842	0.023*	
C3B	−0.14824 (12)	0.53308 (5)	0.88195 (5)	0.01954 (19)	
C4B	−0.17133 (12)	0.59237 (5)	0.85032 (5)	0.0200 (2)	
H4BA	−0.2100	0.6276	0.8641	0.024*	
C5B	−0.13871 (11)	0.60035 (5)	0.79940 (5)	0.01915 (19)	
H5BA	−0.1563	0.6399	0.7774	0.023*	
C6B	−0.07888 (11)	0.54750 (5)	0.78206 (5)	0.01699 (18)	
C7B	0.01755 (11)	0.47587 (4)	0.74094 (5)	0.01714 (18)	
C8B	0.07101 (11)	0.44370 (4)	0.69932 (5)	0.01733 (18)	
C9B	0.14730 (12)	0.47348 (5)	0.66935 (5)	0.01917 (19)	
H9BA	0.1639	0.5187	0.6740	0.023*	
C10B	0.19928 (12)	0.43860 (5)	0.63303 (5)	0.0198 (2)	
H10B	0.2523	0.4603	0.6139	0.024*	
C11B	0.17522 (11)	0.37157 (4)	0.62379 (5)	0.01771 (18)	
C12B	0.10018 (11)	0.34142 (5)	0.65489 (5)	0.0200 (2)	
H12B	0.0835	0.2962	0.6505	0.024*	
C13B	0.05040 (11)	0.37662 (5)	0.69163 (5)	0.01927 (19)	
H13B	0.0009	0.3549	0.7123	0.023*	
C14B	0.33434 (16)	0.36593 (5)	0.57065 (6)	0.0337 (3)	
H14C	0.4211	0.3678	0.6081	0.040*	
H14D	0.3079	0.4108	0.5562	0.040*	
C15B	0.3625 (2)	0.32821 (6)	0.52222 (7)	0.0430 (4)	
H15C	0.2798	0.3310	0.4832	0.052*	
H15D	0.4433	0.3475	0.5159	0.052*	
C16B	0.27266 (15)	0.23460 (6)	0.54554 (6)	0.0325 (3)	
H16C	0.2898	0.1880	0.5551	0.039*	
H16D	0.1911	0.2384	0.5063	0.039*	
C17B	0.23841 (14)	0.26695 (5)	0.59500 (5)	0.0253 (2)	
H17C	0.1505	0.2486	0.5954	0.030*	
H17D	0.3143	0.2582	0.6352	0.030*	
C18B	−0.18227 (14)	0.52350 (5)	0.93653 (5)	0.0261 (2)	
C21B	−0.04038 (11)	0.58836 (4)	0.69036 (5)	0.01778 (18)	
H21C	−0.1275	0.6138	0.6802	0.021*	
H21D	−0.0428	0.5676	0.6525	0.021*	
C22B	0.08605 (11)	0.63358 (4)	0.71524 (5)	0.01756 (18)	
H22C	0.0942	0.6507	0.7553	0.021*	
H22D	0.1724	0.6090	0.7213	0.021*	
C23B	0.00437 (12)	0.74301 (5)	0.68820 (5)	0.01995 (19)	
H23C	0.0618	0.7585	0.7298	0.024*	
H23D	−0.0884	0.7300	0.6873	0.024*	
C24B	−0.01374 (12)	0.79682 (5)	0.64282 (5)	0.0234 (2)	
H24C	−0.0777	0.7823	0.6018	0.028*	
H24D	−0.0572	0.8346	0.6542	0.028*	
C25B	0.18245 (13)	0.76127 (5)	0.62602 (6)	0.0254 (2)	
H25C	0.2731	0.7747	0.6249	0.030*	
H25D	0.1224	0.7449	0.5851	0.030*	
C26B	0.20698 (11)	0.70785 (5)	0.67282 (5)	0.0210 (2)	
H26C	0.2538	0.6708	0.6624	0.025*	
H26D	0.2686	0.7238	0.7137	0.025*	

### Atomic displacement parameters (Å^2^)

**Table:**

	*U*^11^	*U*^22^	*U*^33^	*U*^12^	*U*^13^	*U*^23^
O1A	0.0384 (11)	0.0220 (8)	0.0295 (8)	0.0021 (6)	0.0229 (8)	0.0008 (6)
C19A	0.0407 (12)	0.0294 (9)	0.0318 (9)	0.0027 (8)	0.0273 (9)	0.0008 (7)
C20A	0.0469 (13)	0.0392 (11)	0.0286 (10)	−0.0097 (10)	0.0236 (9)	−0.0034 (8)
O1X	0.043 (3)	0.026 (2)	0.0276 (18)	−0.0087 (16)	0.0228 (19)	−0.0077 (15)
C19X	0.045 (3)	0.033 (2)	0.036 (2)	−0.001 (2)	0.021 (2)	−0.0047 (18)
C20X	0.045 (3)	0.078 (5)	0.044 (3)	−0.007 (3)	0.014 (3)	−0.022 (3)
O4A	0.1043 (11)	0.0402 (6)	0.0580 (7)	−0.0108 (6)	0.0614 (8)	−0.0178 (5)
C18A	0.0456 (8)	0.0327 (6)	0.0333 (7)	−0.0052 (6)	0.0258 (6)	−0.0059 (5)
O2A	0.0288 (5)	0.0295 (4)	0.0368 (5)	0.0026 (4)	0.0156 (4)	−0.0117 (4)
O3A	0.0365 (5)	0.0203 (4)	0.0390 (5)	−0.0063 (3)	0.0232 (4)	−0.0033 (3)
N1A	0.0272 (5)	0.0151 (4)	0.0225 (4)	−0.0002 (3)	0.0111 (4)	−0.0016 (3)
N2A	0.0210 (4)	0.0145 (3)	0.0193 (4)	−0.0011 (3)	0.0097 (3)	−0.0026 (3)
N3A	0.0215 (4)	0.0151 (3)	0.0216 (4)	−0.0006 (3)	0.0111 (4)	−0.0024 (3)
N4A	0.0221 (4)	0.0127 (3)	0.0217 (4)	−0.0011 (3)	0.0101 (4)	−0.0010 (3)
C1A	0.0247 (5)	0.0151 (4)	0.0210 (5)	0.0009 (4)	0.0102 (4)	−0.0014 (3)
C2A	0.0316 (6)	0.0183 (4)	0.0230 (5)	0.0003 (4)	0.0135 (5)	−0.0040 (4)
C3A	0.0288 (6)	0.0235 (5)	0.0238 (5)	0.0018 (4)	0.0146 (5)	−0.0015 (4)
C4A	0.0256 (6)	0.0222 (5)	0.0261 (5)	−0.0028 (4)	0.0140 (5)	−0.0013 (4)
C5A	0.0241 (5)	0.0195 (4)	0.0244 (5)	−0.0030 (4)	0.0121 (4)	−0.0035 (4)
C6A	0.0206 (5)	0.0161 (4)	0.0198 (5)	0.0009 (4)	0.0089 (4)	−0.0017 (3)
C7A	0.0206 (5)	0.0140 (4)	0.0203 (5)	0.0005 (3)	0.0086 (4)	−0.0007 (3)
C8A	0.0196 (5)	0.0143 (4)	0.0197 (5)	−0.0005 (3)	0.0071 (4)	−0.0011 (3)
C9A	0.0207 (5)	0.0126 (4)	0.0205 (5)	0.0004 (3)	0.0075 (4)	−0.0006 (3)
C10A	0.0198 (5)	0.0143 (4)	0.0205 (5)	0.0006 (3)	0.0085 (4)	−0.0017 (3)
C11A	0.0177 (5)	0.0135 (4)	0.0183 (4)	−0.0007 (3)	0.0059 (4)	−0.0008 (3)
C12A	0.0205 (5)	0.0128 (4)	0.0246 (5)	0.0019 (3)	0.0092 (4)	0.0006 (3)
C13A	0.0200 (5)	0.0145 (4)	0.0241 (5)	0.0009 (4)	0.0092 (4)	−0.0018 (3)
C14A	0.0275 (6)	0.0173 (4)	0.0258 (5)	0.0011 (4)	0.0145 (5)	−0.0007 (4)
C15A	0.0422 (7)	0.0185 (4)	0.0314 (6)	−0.0037 (5)	0.0229 (6)	−0.0010 (4)
C16A	0.0300 (6)	0.0167 (4)	0.0274 (5)	−0.0036 (4)	0.0124 (5)	−0.0013 (4)
C17A	0.0342 (6)	0.0143 (4)	0.0297 (6)	−0.0053 (4)	0.0167 (5)	−0.0038 (4)
C21A	0.0208 (5)	0.0156 (4)	0.0188 (5)	−0.0014 (4)	0.0083 (4)	−0.0040 (3)
C22A	0.0232 (5)	0.0155 (4)	0.0179 (4)	0.0012 (4)	0.0090 (4)	−0.0015 (3)
C23A	0.0340 (6)	0.0153 (4)	0.0316 (6)	0.0028 (4)	0.0166 (5)	0.0009 (4)
C24A	0.0316 (7)	0.0191 (5)	0.0438 (7)	−0.0012 (4)	0.0168 (6)	−0.0087 (5)
C25A	0.0414 (8)	0.0364 (6)	0.0279 (6)	0.0055 (6)	0.0186 (6)	−0.0062 (5)
C26A	0.0347 (7)	0.0237 (5)	0.0270 (6)	−0.0007 (5)	0.0197 (5)	−0.0013 (4)
O1B	0.0306 (6)	0.0160 (4)	0.0227 (5)	0.0003 (4)	0.0144 (5)	−0.0005 (3)
C19B	0.0305 (7)	0.0211 (5)	0.0170 (6)	0.0020 (5)	0.0097 (5)	−0.0001 (5)
C20B	0.0313 (8)	0.0348 (7)	0.0234 (7)	0.0024 (6)	0.0139 (6)	0.0008 (5)
O1Y	0.022 (4)	0.021 (3)	0.023 (3)	0.004 (3)	0.015 (3)	0.002 (2)
C19Y	0.0305 (7)	0.0211 (5)	0.0170 (6)	0.0020 (5)	0.0097 (5)	−0.0001 (5)
C20Y	0.087 (12)	0.027 (6)	0.061 (10)	−0.015 (6)	0.041 (10)	−0.009 (7)
O2B	0.0342 (5)	0.0143 (3)	0.0374 (5)	−0.0017 (3)	0.0182 (4)	0.0026 (3)
O3B	0.0621 (7)	0.0212 (4)	0.0414 (5)	0.0092 (4)	0.0370 (5)	0.0028 (4)
O4B	0.0762 (8)	0.0163 (4)	0.0424 (5)	0.0007 (4)	0.0433 (6)	0.0019 (3)
N1B	0.0234 (5)	0.0133 (3)	0.0228 (4)	−0.0002 (3)	0.0119 (4)	0.0011 (3)
N2B	0.0207 (4)	0.0127 (3)	0.0193 (4)	−0.0002 (3)	0.0095 (3)	0.0011 (3)
N3B	0.0187 (4)	0.0117 (3)	0.0209 (4)	0.0008 (3)	0.0096 (3)	0.0019 (3)
N4B	0.0262 (5)	0.0143 (3)	0.0201 (4)	0.0020 (3)	0.0093 (4)	−0.0020 (3)
C1B	0.0196 (5)	0.0128 (4)	0.0212 (5)	−0.0010 (3)	0.0091 (4)	0.0007 (3)
C2B	0.0234 (5)	0.0131 (4)	0.0232 (5)	−0.0014 (4)	0.0114 (4)	0.0010 (3)
C3B	0.0247 (5)	0.0143 (4)	0.0224 (5)	−0.0013 (4)	0.0123 (4)	0.0001 (3)
C4B	0.0230 (5)	0.0153 (4)	0.0241 (5)	0.0015 (4)	0.0119 (4)	0.0010 (3)
C5B	0.0223 (5)	0.0139 (4)	0.0227 (5)	0.0016 (4)	0.0103 (4)	0.0024 (3)
C6B	0.0184 (5)	0.0149 (4)	0.0185 (4)	−0.0013 (3)	0.0080 (4)	0.0002 (3)
C7B	0.0193 (5)	0.0126 (4)	0.0206 (5)	−0.0014 (3)	0.0089 (4)	−0.0003 (3)
C8B	0.0196 (5)	0.0129 (4)	0.0206 (5)	−0.0009 (3)	0.0089 (4)	−0.0010 (3)
C9B	0.0252 (5)	0.0124 (4)	0.0227 (5)	−0.0020 (4)	0.0124 (4)	−0.0017 (3)
C10B	0.0273 (6)	0.0135 (4)	0.0219 (5)	−0.0011 (4)	0.0132 (4)	−0.0006 (3)
C11B	0.0206 (5)	0.0137 (4)	0.0177 (4)	0.0014 (3)	0.0062 (4)	−0.0012 (3)
C12B	0.0226 (5)	0.0120 (4)	0.0253 (5)	−0.0013 (4)	0.0093 (4)	−0.0014 (3)
C13B	0.0217 (5)	0.0126 (4)	0.0252 (5)	−0.0011 (3)	0.0110 (4)	0.0002 (3)
C14B	0.0572 (9)	0.0205 (5)	0.0409 (7)	−0.0025 (5)	0.0381 (7)	−0.0042 (5)
C15B	0.0853 (12)	0.0207 (5)	0.0445 (8)	0.0091 (6)	0.0486 (9)	0.0035 (5)
C16B	0.0480 (8)	0.0197 (5)	0.0316 (6)	0.0025 (5)	0.0174 (6)	−0.0071 (4)
C17B	0.0346 (6)	0.0143 (4)	0.0314 (6)	0.0005 (4)	0.0177 (5)	−0.0035 (4)
C18B	0.0393 (7)	0.0170 (4)	0.0294 (6)	0.0015 (4)	0.0213 (5)	0.0005 (4)
C21B	0.0208 (5)	0.0135 (4)	0.0190 (5)	−0.0005 (3)	0.0078 (4)	0.0027 (3)
C22B	0.0193 (5)	0.0135 (4)	0.0192 (5)	0.0001 (3)	0.0067 (4)	0.0021 (3)
C23B	0.0230 (5)	0.0148 (4)	0.0241 (5)	0.0029 (4)	0.0114 (4)	0.0013 (3)
C24B	0.0264 (6)	0.0152 (4)	0.0284 (5)	0.0031 (4)	0.0105 (5)	0.0028 (4)
C25B	0.0307 (6)	0.0194 (4)	0.0318 (6)	−0.0014 (4)	0.0185 (5)	0.0031 (4)
C26B	0.0203 (5)	0.0178 (4)	0.0263 (5)	−0.0007 (4)	0.0108 (4)	0.0008 (4)

### Geometric parameters (Å, °)

**Table:**

O1A—C18A	1.298 (2)	O1B—C18B	1.3484 (13)
O1A—C19A	1.451 (2)	O1B—C19B	1.4520 (16)
C19A—C20A	1.495 (3)	C19B—C20B	1.503 (2)
C19A—H19A	0.9900	C19B—H19E	0.9900
C19A—H19B	0.9900	C19B—H19F	0.9900
C20A—H20A	0.9800	C20B—H20G	0.9800
C20A—H20B	0.9800	C20B—H20H	0.9800
C20A—H20C	0.9800	C20B—H20I	0.9800
O1X—C19X	1.475 (6)	O1Y—C19Y	1.459 (10)
O1X—C18A	1.517 (5)	O1Y—C18B	1.478 (6)
C19X—C20X	1.473 (8)	C19Y—C20Y	1.461 (14)
C19X—H19C	0.9900	C19Y—H19G	0.9900
C19X—H19D	0.9900	C19Y—H19H	0.9900
C20X—H20D	0.9800	C20Y—H20J	0.9800
C20X—H20E	0.9800	C20Y—H20K	0.9800
C20X—H20F	0.9800	C20Y—H20L	0.9800
O4A—C18A	1.2033 (15)	O2B—C24B	1.4251 (14)
C18A—C3A	1.4852 (16)	O2B—C25B	1.4290 (14)
O2A—C24A	1.4208 (16)	O3B—C16B	1.4164 (17)
O2A—C25A	1.4243 (16)	O3B—C15B	1.4283 (14)
O3A—C16A	1.4181 (14)	O4B—C18B	1.2077 (13)
O3A—C15A	1.4305 (14)	N1B—C7B	1.3220 (13)
N1A—C7A	1.3247 (13)	N1B—C1B	1.3830 (13)
N1A—C1A	1.3872 (14)	N2B—C6B	1.3827 (13)
N2A—C6A	1.3780 (13)	N2B—C7B	1.3871 (12)
N2A—C7A	1.3862 (13)	N2B—C21B	1.4627 (12)
N2A—C21A	1.4602 (12)	N3B—C22B	1.4583 (12)
N3A—C22A	1.4575 (12)	N3B—C26B	1.4624 (13)
N3A—C23A	1.4641 (14)	N3B—C23B	1.4645 (13)
N3A—C26A	1.4648 (14)	N4B—C11B	1.3942 (13)
N4A—C11A	1.3988 (13)	N4B—C17B	1.4629 (13)
N4A—C14A	1.4622 (13)	N4B—C14B	1.4637 (15)
N4A—C17A	1.4721 (13)	C1B—C2B	1.3910 (14)
C1A—C2A	1.3936 (14)	C1B—C6B	1.4093 (13)
C1A—C6A	1.4083 (14)	C2B—C3B	1.3912 (14)
C2A—C3A	1.3911 (16)	C2B—H2BA	0.9500
C2A—H2AA	0.9500	C3B—C4B	1.4101 (14)
C3A—C4A	1.4113 (15)	C3B—C18B	1.4849 (15)
C4A—C5A	1.3854 (15)	C4B—C5B	1.3880 (14)
C4A—H4AA	0.9500	C4B—H4BA	0.9500
C5A—C6A	1.3943 (14)	C5B—C6B	1.3959 (14)
C5A—H5AA	0.9500	C5B—H5BA	0.9500
C7A—C8A	1.4676 (14)	C7B—C8B	1.4682 (14)
C8A—C9A	1.3969 (14)	C8B—C9B	1.3965 (14)
C8A—C13A	1.4051 (14)	C8B—C13B	1.4050 (13)
C9A—C10A	1.3922 (14)	C9B—C10B	1.3852 (14)
C9A—H9AA	0.9500	C9B—H9BA	0.9500
C10A—C11A	1.4099 (13)	C10B—C11B	1.4108 (13)
C10A—H10A	0.9500	C10B—H10B	0.9500
C11A—C12A	1.4077 (14)	C11B—C12B	1.4096 (14)
C12A—C13A	1.3796 (14)	C12B—C13B	1.3822 (14)
C12A—H12A	0.9500	C12B—H12B	0.9500
C13A—H13A	0.9500	C13B—H13B	0.9500
C14A—C15A	1.5167 (15)	C14B—C15B	1.5106 (16)
C14A—H14A	0.9900	C14B—H14C	0.9900
C14A—H14B	0.9900	C14B—H14D	0.9900
C15A—H15A	0.9900	C15B—H15C	0.9900
C15A—H15B	0.9900	C15B—H15D	0.9900
C16A—C17A	1.5084 (15)	C16B—C17B	1.5108 (16)
C16A—H16A	0.9900	C16B—H16C	0.9900
C16A—H16B	0.9900	C16B—H16D	0.9900
C17A—H17A	0.9900	C17B—H17C	0.9900
C17A—H17B	0.9900	C17B—H17D	0.9900
C21A—C22A	1.5298 (14)	C21B—C22B	1.5294 (14)
C21A—H21A	0.9900	C21B—H21C	0.9900
C21A—H21B	0.9900	C21B—H21D	0.9900
C22A—H22A	0.9900	C22B—H22C	0.9900
C22A—H22B	0.9900	C22B—H22D	0.9900
C23A—C24A	1.5120 (16)	C23B—C24B	1.5108 (14)
C23A—H23A	0.9900	C23B—H23C	0.9900
C23A—H23B	0.9900	C23B—H23D	0.9900
C24A—H24A	0.9900	C24B—H24C	0.9900
C24A—H24B	0.9900	C24B—H24D	0.9900
C25A—C26A	1.5125 (16)	C25B—C26B	1.5185 (15)
C25A—H25A	0.9900	C25B—H25C	0.9900
C25A—H25B	0.9900	C25B—H25D	0.9900
C26A—H26A	0.9900	C26B—H26C	0.9900
C26A—H26B	0.9900	C26B—H26D	0.9900
			
C18A—O1A—C19A	115.53 (16)	C18B—O1B—C19B	115.73 (9)
O1A—C19A—C20A	109.17 (16)	O1B—C19B—C20B	111.26 (13)
O1A—C19A—H19A	109.8	O1B—C19B—H19E	109.4
C20A—C19A—H19A	109.8	C20B—C19B—H19E	109.4
O1A—C19A—H19B	109.8	O1B—C19B—H19F	109.4
C20A—C19A—H19B	109.8	C20B—C19B—H19F	109.4
H19A—C19A—H19B	108.3	H19E—C19B—H19F	108.0
C19X—O1X—C18A	116.1 (3)	C19Y—O1Y—C18B	114.4 (6)
C20X—C19X—O1X	108.0 (4)	O1Y—C19Y—C20Y	111.1 (11)
C20X—C19X—H19C	110.1	O1Y—C19Y—H19G	109.4
O1X—C19X—H19C	110.1	C20Y—C19Y—H19G	109.4
C20X—C19X—H19D	110.1	O1Y—C19Y—H19H	109.4
O1X—C19X—H19D	110.1	C20Y—C19Y—H19H	109.4
H19C—C19X—H19D	108.4	H19G—C19Y—H19H	108.0
C19X—C20X—H20D	109.5	C19Y—C20Y—H20J	109.5
C19X—C20X—H20E	109.5	C19Y—C20Y—H20K	109.5
H20D—C20X—H20E	109.5	H20J—C20Y—H20K	109.5
C19X—C20X—H20F	109.5	C19Y—C20Y—H20L	109.5
H20D—C20X—H20F	109.5	H20J—C20Y—H20L	109.5
H20E—C20X—H20F	109.5	H20K—C20Y—H20L	109.5
O4A—C18A—O1A	120.02 (14)	C24B—O2B—C25B	109.94 (8)
O4A—C18A—C3A	123.89 (12)	C16B—O3B—C15B	108.41 (11)
O1A—C18A—C3A	115.71 (13)	C7B—N1B—C1B	105.01 (8)
O4A—C18A—O1X	126.49 (19)	C6B—N2B—C7B	106.24 (8)
C3A—C18A—O1X	107.76 (18)	C6B—N2B—C21B	123.92 (8)
C24A—O2A—C25A	110.06 (9)	C7B—N2B—C21B	129.83 (8)
C16A—O3A—C15A	108.79 (9)	C22B—N3B—C26B	112.98 (8)
C7A—N1A—C1A	104.94 (8)	C22B—N3B—C23B	111.44 (8)
C6A—N2A—C7A	106.55 (8)	C26B—N3B—C23B	108.81 (8)
C6A—N2A—C21A	123.95 (8)	C11B—N4B—C17B	116.91 (9)
C7A—N2A—C21A	129.41 (8)	C11B—N4B—C14B	117.12 (8)
C22A—N3A—C23A	111.83 (8)	C17B—N4B—C14B	113.09 (9)
C22A—N3A—C26A	111.49 (8)	N1B—C1B—C2B	129.38 (9)
C23A—N3A—C26A	108.96 (9)	N1B—C1B—C6B	110.34 (9)
C11A—N4A—C14A	117.01 (8)	C2B—C1B—C6B	120.27 (9)
C11A—N4A—C17A	116.04 (8)	C1B—C2B—C3B	117.89 (9)
C14A—N4A—C17A	112.82 (9)	C1B—C2B—H2BA	121.1
N1A—C1A—C2A	129.88 (9)	C3B—C2B—H2BA	121.1
N1A—C1A—C6A	110.21 (9)	C2B—C3B—C4B	121.30 (9)
C2A—C1A—C6A	119.91 (10)	C2B—C3B—C18B	116.41 (9)
C3A—C2A—C1A	118.05 (10)	C4B—C3B—C18B	122.28 (9)
C3A—C2A—H2AA	121.0	C5B—C4B—C3B	121.39 (9)
C1A—C2A—H2AA	121.0	C5B—C4B—H4BA	119.3
C2A—C3A—C4A	121.25 (10)	C3B—C4B—H4BA	119.3
C2A—C3A—C18A	116.56 (10)	C4B—C5B—C6B	116.83 (9)
C4A—C3A—C18A	122.16 (10)	C4B—C5B—H5BA	121.6
C5A—C4A—C3A	121.37 (10)	C6B—C5B—H5BA	121.6
C5A—C4A—H4AA	119.3	N2B—C6B—C5B	132.29 (9)
C3A—C4A—H4AA	119.3	N2B—C6B—C1B	105.45 (8)
C4A—C5A—C6A	116.83 (10)	C5B—C6B—C1B	122.26 (9)
C4A—C5A—H5AA	121.6	N1B—C7B—N2B	112.95 (9)
C6A—C5A—H5AA	121.6	N1B—C7B—C8B	121.16 (9)
N2A—C6A—C5A	131.84 (9)	N2B—C7B—C8B	125.80 (9)
N2A—C6A—C1A	105.57 (9)	C9B—C8B—C13B	117.17 (9)
C5A—C6A—C1A	122.57 (9)	C9B—C8B—C7B	125.50 (9)
N1A—C7A—N2A	112.74 (9)	C13B—C8B—C7B	117.23 (9)
N1A—C7A—C8A	122.08 (9)	C10B—C9B—C8B	121.53 (9)
N2A—C7A—C8A	125.06 (9)	C10B—C9B—H9BA	119.2
C9A—C8A—C13A	117.35 (9)	C8B—C9B—H9BA	119.2
C9A—C8A—C7A	124.98 (9)	C9B—C10B—C11B	121.41 (9)
C13A—C8A—C7A	117.60 (9)	C9B—C10B—H10B	119.3
C10A—C9A—C8A	121.31 (9)	C11B—C10B—H10B	119.3
C10A—C9A—H9AA	119.3	N4B—C11B—C12B	121.47 (9)
C8A—C9A—H9AA	119.3	N4B—C11B—C10B	121.57 (9)
C9A—C10A—C11A	121.22 (9)	C12B—C11B—C10B	116.95 (9)
C9A—C10A—H10A	119.4	C13B—C12B—C11B	121.06 (9)
C11A—C10A—H10A	119.4	C13B—C12B—H12B	119.5
N4A—C11A—C12A	120.55 (8)	C11B—C12B—H12B	119.5
N4A—C11A—C10A	122.32 (9)	C12B—C13B—C8B	121.84 (9)
C12A—C11A—C10A	117.11 (9)	C12B—C13B—H13B	119.1
C13A—C12A—C11A	121.23 (9)	C8B—C13B—H13B	119.1
C13A—C12A—H12A	119.4	N4B—C14B—C15B	111.95 (11)
C11A—C12A—H12A	119.4	N4B—C14B—H14C	109.2
C12A—C13A—C8A	121.76 (9)	C15B—C14B—H14C	109.2
C12A—C13A—H13A	119.1	N4B—C14B—H14D	109.2
C8A—C13A—H13A	119.1	C15B—C14B—H14D	109.2
N4A—C14A—C15A	111.56 (9)	H14C—C14B—H14D	107.9
N4A—C14A—H14A	109.3	O3B—C15B—C14B	111.26 (10)
C15A—C14A—H14A	109.3	O3B—C15B—H15C	109.4
N4A—C14A—H14B	109.3	C14B—C15B—H15C	109.4
C15A—C14A—H14B	109.3	O3B—C15B—H15D	109.4
H14A—C14A—H14B	108.0	C14B—C15B—H15D	109.4
O3A—C15A—C14A	111.86 (9)	H15C—C15B—H15D	108.0
O3A—C15A—H15A	109.2	O3B—C16B—C17B	112.55 (10)
C14A—C15A—H15A	109.2	O3B—C16B—H16C	109.1
O3A—C15A—H15B	109.2	C17B—C16B—H16C	109.1
C14A—C15A—H15B	109.2	O3B—C16B—H16D	109.1
H15A—C15A—H15B	107.9	C17B—C16B—H16D	109.1
O3A—C16A—C17A	111.67 (9)	H16C—C16B—H16D	107.8
O3A—C16A—H16A	109.3	N4B—C17B—C16B	111.15 (9)
C17A—C16A—H16A	109.3	N4B—C17B—H17C	109.4
O3A—C16A—H16B	109.3	C16B—C17B—H17C	109.4
C17A—C16A—H16B	109.3	N4B—C17B—H17D	109.4
H16A—C16A—H16B	107.9	C16B—C17B—H17D	109.4
N4A—C17A—C16A	111.41 (9)	H17C—C17B—H17D	108.0
N4A—C17A—H17A	109.3	O4B—C18B—O1B	123.17 (10)
C16A—C17A—H17A	109.3	O4B—C18B—O1Y	117.7 (3)
N4A—C17A—H17B	109.3	O4B—C18B—C3B	124.04 (10)
C16A—C17A—H17B	109.3	O1B—C18B—C3B	112.70 (9)
H17A—C17A—H17B	108.0	O1Y—C18B—C3B	108.5 (2)
N2A—C21A—C22A	109.96 (8)	N2B—C21B—C22B	110.99 (8)
N2A—C21A—H21A	109.7	N2B—C21B—H21C	109.4
C22A—C21A—H21A	109.7	C22B—C21B—H21C	109.4
N2A—C21A—H21B	109.7	N2B—C21B—H21D	109.4
C22A—C21A—H21B	109.7	C22B—C21B—H21D	109.4
H21A—C21A—H21B	108.2	H21C—C21B—H21D	108.0
N3A—C22A—C21A	111.70 (8)	N3B—C22B—C21B	111.15 (8)
N3A—C22A—H22A	109.3	N3B—C22B—H22C	109.4
C21A—C22A—H22A	109.3	C21B—C22B—H22C	109.4
N3A—C22A—H22B	109.3	N3B—C22B—H22D	109.4
C21A—C22A—H22B	109.3	C21B—C22B—H22D	109.4
H22A—C22A—H22B	107.9	H22C—C22B—H22D	108.0
N3A—C23A—C24A	110.02 (10)	N3B—C23B—C24B	110.39 (8)
N3A—C23A—H23A	109.7	N3B—C23B—H23C	109.6
C24A—C23A—H23A	109.7	C24B—C23B—H23C	109.6
N3A—C23A—H23B	109.7	N3B—C23B—H23D	109.6
C24A—C23A—H23B	109.7	C24B—C23B—H23D	109.6
H23A—C23A—H23B	108.2	H23C—C23B—H23D	108.1
O2A—C24A—C23A	111.05 (9)	O2B—C24B—C23B	111.75 (9)
O2A—C24A—H24A	109.4	O2B—C24B—H24C	109.3
C23A—C24A—H24A	109.4	C23B—C24B—H24C	109.3
O2A—C24A—H24B	109.4	O2B—C24B—H24D	109.3
C23A—C24A—H24B	109.4	C23B—C24B—H24D	109.3
H24A—C24A—H24B	108.0	H24C—C24B—H24D	107.9
O2A—C25A—C26A	111.77 (10)	O2B—C25B—C26B	110.73 (9)
O2A—C25A—H25A	109.3	O2B—C25B—H25C	109.5
C26A—C25A—H25A	109.3	C26B—C25B—H25C	109.5
O2A—C25A—H25B	109.3	O2B—C25B—H25D	109.5
C26A—C25A—H25B	109.3	C26B—C25B—H25D	109.5
H25A—C25A—H25B	107.9	H25C—C25B—H25D	108.1
N3A—C26A—C25A	110.20 (10)	N3B—C26B—C25B	109.40 (9)
N3A—C26A—H26A	109.6	N3B—C26B—H26C	109.8
C25A—C26A—H26A	109.6	C25B—C26B—H26C	109.8
N3A—C26A—H26B	109.6	N3B—C26B—H26D	109.8
C25A—C26A—H26B	109.6	C25B—C26B—H26D	109.8
H26A—C26A—H26B	108.1	H26C—C26B—H26D	108.2
			
C18A—O1A—C19A—C20A	−173.2 (2)	C18B—O1B—C19B—C20B	−84.92 (15)
C18A—O1X—C19X—C20X	86.8 (6)	C18B—O1Y—C19Y—C20Y	62.4 (10)
C19A—O1A—C18A—O4A	−2.7 (3)	C7B—N1B—C1B—C2B	178.19 (11)
C19A—O1A—C18A—C3A	−175.87 (15)	C7B—N1B—C1B—C6B	−0.64 (12)
C19A—O1A—C18A—O1X	111.3 (7)	N1B—C1B—C2B—C3B	178.37 (11)
C19X—O1X—C18A—O4A	10.8 (5)	C6B—C1B—C2B—C3B	−2.90 (16)
C19X—O1X—C18A—O1A	−69.1 (6)	C1B—C2B—C3B—C4B	1.96 (17)
C19X—O1X—C18A—C3A	175.6 (3)	C1B—C2B—C3B—C18B	−177.72 (10)
C7A—N1A—C1A—C2A	−179.42 (12)	C2B—C3B—C4B—C5B	0.30 (17)
C7A—N1A—C1A—C6A	−0.30 (12)	C18B—C3B—C4B—C5B	179.96 (11)
N1A—C1A—C2A—C3A	179.53 (11)	C3B—C4B—C5B—C6B	−1.57 (16)
C6A—C1A—C2A—C3A	0.48 (17)	C7B—N2B—C6B—C5B	179.54 (11)
C1A—C2A—C3A—C4A	−1.01 (18)	C21B—N2B—C6B—C5B	0.53 (18)
C1A—C2A—C3A—C18A	−179.19 (11)	C7B—N2B—C6B—C1B	−0.75 (11)
O4A—C18A—C3A—C2A	5.8 (2)	C21B—N2B—C6B—C1B	−179.76 (9)
O1A—C18A—C3A—C2A	178.74 (15)	C4B—C5B—C6B—N2B	−179.73 (11)
O1X—C18A—C3A—C2A	−159.5 (2)	C4B—C5B—C6B—C1B	0.60 (16)
O4A—C18A—C3A—C4A	−172.36 (15)	N1B—C1B—C6B—N2B	0.88 (12)
O1A—C18A—C3A—C4A	0.6 (2)	C2B—C1B—C6B—N2B	−178.08 (10)
O1X—C18A—C3A—C4A	22.3 (3)	N1B—C1B—C6B—C5B	−179.37 (10)
C2A—C3A—C4A—C5A	0.06 (18)	C2B—C1B—C6B—C5B	1.67 (16)
C18A—C3A—C4A—C5A	178.14 (12)	C1B—N1B—C7B—N2B	0.16 (12)
C3A—C4A—C5A—C6A	1.39 (17)	C1B—N1B—C7B—C8B	−176.65 (9)
C7A—N2A—C6A—C5A	178.15 (12)	C6B—N2B—C7B—N1B	0.39 (12)
C21A—N2A—C6A—C5A	−5.01 (18)	C21B—N2B—C7B—N1B	179.32 (10)
C7A—N2A—C6A—C1A	−0.03 (11)	C6B—N2B—C7B—C8B	177.01 (10)
C21A—N2A—C6A—C1A	176.80 (9)	C21B—N2B—C7B—C8B	−4.05 (17)
C4A—C5A—C6A—N2A	−179.86 (11)	N1B—C7B—C8B—C9B	−147.51 (11)
C4A—C5A—C6A—C1A	−1.94 (17)	N2B—C7B—C8B—C9B	36.12 (17)
N1A—C1A—C6A—N2A	0.21 (12)	N1B—C7B—C8B—C13B	28.76 (15)
C2A—C1A—C6A—N2A	179.42 (10)	N2B—C7B—C8B—C13B	−147.61 (11)
N1A—C1A—C6A—C5A	−178.19 (10)	C13B—C8B—C9B—C10B	0.67 (16)
C2A—C1A—C6A—C5A	1.03 (17)	C7B—C8B—C9B—C10B	176.94 (10)
C1A—N1A—C7A—N2A	0.28 (12)	C8B—C9B—C10B—C11B	1.19 (17)
C1A—N1A—C7A—C8A	176.39 (10)	C17B—N4B—C11B—C12B	−22.10 (15)
C6A—N2A—C7A—N1A	−0.16 (12)	C14B—N4B—C11B—C12B	−161.06 (11)
C21A—N2A—C7A—N1A	−176.76 (10)	C17B—N4B—C11B—C10B	158.50 (11)
C6A—N2A—C7A—C8A	−176.13 (10)	C14B—N4B—C11B—C10B	19.54 (15)
C21A—N2A—C7A—C8A	7.27 (17)	C9B—C10B—C11B—N4B	177.27 (10)
N1A—C7A—C8A—C9A	139.33 (11)	C9B—C10B—C11B—C12B	−2.15 (16)
N2A—C7A—C8A—C9A	−45.05 (16)	N4B—C11B—C12B—C13B	−178.12 (10)
N1A—C7A—C8A—C13A	−37.46 (15)	C10B—C11B—C12B—C13B	1.30 (16)
N2A—C7A—C8A—C13A	138.16 (11)	C11B—C12B—C13B—C8B	0.53 (17)
C13A—C8A—C9A—C10A	0.33 (16)	C9B—C8B—C13B—C12B	−1.52 (16)
C7A—C8A—C9A—C10A	−176.47 (10)	C7B—C8B—C13B—C12B	−178.11 (10)
C8A—C9A—C10A—C11A	−1.16 (16)	C11B—N4B—C14B—C15B	−172.34 (11)
C14A—N4A—C11A—C12A	174.99 (10)	C17B—N4B—C14B—C15B	47.20 (15)
C17A—N4A—C11A—C12A	37.80 (14)	C16B—O3B—C15B—C14B	61.40 (16)
C14A—N4A—C11A—C10A	−6.41 (15)	N4B—C14B—C15B—O3B	−54.87 (18)
C17A—N4A—C11A—C10A	−143.60 (11)	C15B—O3B—C16B—C17B	−61.80 (14)
C9A—C10A—C11A—N4A	−177.58 (10)	C11B—N4B—C17B—C16B	173.03 (10)
C9A—C10A—C11A—C12A	1.07 (15)	C14B—N4B—C17B—C16B	−46.42 (14)
N4A—C11A—C12A—C13A	178.48 (10)	O3B—C16B—C17B—N4B	54.58 (14)
C10A—C11A—C12A—C13A	−0.19 (16)	C19B—O1B—C18B—O4B	−1.2 (2)
C11A—C12A—C13A—C8A	−0.63 (17)	C19B—O1B—C18B—O1Y	91.5 (4)
C9A—C8A—C13A—C12A	0.56 (16)	C19B—O1B—C18B—C3B	−177.84 (11)
C7A—C8A—C13A—C12A	177.60 (10)	C19Y—O1Y—C18B—O4B	26.3 (8)
C11A—N4A—C14A—C15A	174.35 (9)	C19Y—O1Y—C18B—O1B	−83.0 (7)
C17A—N4A—C14A—C15A	−47.14 (13)	C19Y—O1Y—C18B—C3B	173.6 (6)
C16A—O3A—C15A—C14A	−60.93 (13)	C2B—C3B—C18B—O4B	−16.55 (19)
N4A—C14A—C15A—O3A	54.02 (14)	C4B—C3B—C18B—O4B	163.78 (13)
C15A—O3A—C16A—C17A	61.79 (12)	C2B—C3B—C18B—O1B	160.06 (11)
C11A—N4A—C17A—C16A	−173.12 (9)	C4B—C3B—C18B—O1B	−19.62 (17)
C14A—N4A—C17A—C16A	47.94 (13)	C2B—C3B—C18B—O1Y	−161.3 (3)
O3A—C16A—C17A—N4A	−55.71 (13)	C4B—C3B—C18B—O1Y	19.0 (4)
C6A—N2A—C21A—C22A	−75.87 (12)	C6B—N2B—C21B—C22B	83.50 (12)
C7A—N2A—C21A—C22A	100.20 (12)	C7B—N2B—C21B—C22B	−95.26 (12)
C23A—N3A—C22A—C21A	−154.95 (9)	C26B—N3B—C22B—C21B	−145.67 (9)
C26A—N3A—C22A—C21A	82.78 (11)	C23B—N3B—C22B—C21B	91.46 (10)
N2A—C21A—C22A—N3A	−177.11 (8)	N2B—C21B—C22B—N3B	−174.05 (8)
C22A—N3A—C23A—C24A	178.76 (9)	C22B—N3B—C23B—C24B	−177.62 (9)
C26A—N3A—C23A—C24A	−57.54 (12)	C26B—N3B—C23B—C24B	57.15 (11)
C25A—O2A—C24A—C23A	−58.52 (13)	C25B—O2B—C24B—C23B	57.38 (12)
N3A—C23A—C24A—O2A	59.31 (14)	N3B—C23B—C24B—O2B	−57.12 (12)
C24A—O2A—C25A—C26A	57.69 (14)	C24B—O2B—C25B—C26B	−58.85 (12)
C22A—N3A—C26A—C25A	−179.66 (10)	C22B—N3B—C26B—C25B	177.08 (8)
C23A—N3A—C26A—C25A	56.43 (13)	C23B—N3B—C26B—C25B	−58.59 (11)
O2A—C25A—C26A—N3A	−57.24 (14)	O2B—C25B—C26B—N3B	60.28 (12)

### Hydrogen-bond geometry (Å, °)

**Table:**

*D*—H···*A*	*D*—H	H···*A*	*D*···*A*	*D*—H···*A*
C14B—H14D···O4A^i^	0.99	2.39	3.1322 (17)	132
C16B—H16C···O1B^ii^	0.99	2.42	3.2944 (15)	147
C17B—H17C···O3A^iii^	0.99	2.60	3.4963 (18)	151
C23B—H23D···O2A^iv^	0.99	2.53	3.3839 (16)	144
C24B—H24D···N1B^v^	0.99	2.61	3.4668 (14)	145
C25B—H25C···N3A	0.99	2.61	3.5189 (18)	153

Symmetry codes: (i) *x*, −*y*+3/2, *z*−1/2; (ii) −*x*, *y*−1/2, −*z*+3/2; (iii) *x*−1, *y*−1, *z*; (iv) *x*−1, *y*, *z*; (v) −*x*, *y*+1/2, −*z*+3/2.

## References

[bb1] Bruker (2009). *APEX2*, *SAINT* and *SADABS* Bruker AXS Inc., Madison, Wisconsin, USA.

[bb2] Cosier, J. & Glazer, A. M. (1986). *J. Appl. Cryst.* **19**, 105–107.

[bb3] Haugwitz, R. D. (1982). *J. Med. Chem.* **25**, 969–974. 10.1021/jm00350a017 7120286

[bb4] Hisano, T. (1982). *Chem. Pharm. Bull.* **30**, 2996–3004.

[bb5] Sheldrick, G. M. (2008). *Acta Cryst.* A**64**, 112–122. 10.1107/S0108767307043930 18156677

[bb6] Spek, A. L. (2009). *Acta Cryst.* D**65**, 148–155. 10.1107/S090744490804362X PMC2631630 19171970

[bb7] Vijaya, B. R., Rajeev, K. S., Varadaraj, B. G. & Gautham, G. S. (2009). *Asian J. Res. Chem.* **2**, 162–167.

